# Local Anaesthetic Thoracoscopy for Pleural Effusion—A Narrative Review

**DOI:** 10.3390/healthcare10101978

**Published:** 2022-10-09

**Authors:** Dana Li, Karl Jackson, Rakesh Panchal, Avinash Aujayeb

**Affiliations:** 1 Institute for Lung Health, University Hospitals of Leicester NHS Trust, Glenfield Hospital, Groby Road, Leicester LE3 9QP, UK; 2 Respiratory Department, Northumbria Health Care NHS Foundation Trust, Care of Gail Hewitt, Newcastle NE23 6NZ, UK

**Keywords:** medical thoracoscopy, local anaesthetic thoracoscopy, pleural effusion

## Abstract

The incidence of pleural disease is increasing, and interventions are crucial in this subspecialist area of respiratory medicine. One of the cornerstones of pleural effusion investigation and management is medical, which is also known as local anaesthetic thoracoscopy. This allows fluid drainage, biopsy for diagnosis and preventative measures for further fluid potential build-up. This article summarises the evidence around this procedure through a narrative review of the available evidence.

## 1. Introduction

Thoracoscopy, meaning examination of the thoracic cavity, has been a technique used since the 19th century. Hans-Christian Jacobaeus is widely credited with first performing and publishing his technique of using a cystoscope to insufflate air into the thoracic cavity and diagnose tuberculous pleural adhesions in 1910 [1]. The French, however, may have been the first to have conceptualised the idea of “thoracoscopie”, with medical texts such as the *Complement du Dictionnaire de l’Academic Française* using the term as early as 1842 [2]. Furthermore, in 1865, the *British Medical Journal* published a report whereby Sir Francis Richard Cruise, an Irish endoscopist, used regular direct endoscopic inspection of the pleura to monitor a patient’s protracted recovery from pleural infection. Together with Samuel Gordon, an Irish internist at the time, Cruise used a modified endoscope that had been created by A J Desormeaux. Furthermore, Carlo Forlanini, in Pavia, Italy, used a similar method in 1882 to create an artificial pneumothorax and induce lung collapse, a treatment that was thought to be beneficial in pulmonary tuberculosis [2]. Direct visualisation of the pleura enables biopsies to be taken from the parietal pleura and pleural-based abnormalities such as nodules, increasing diagnostic yield, particularly in malignancy [3]. At the same time, any fluid can be drained offering symptomatic relief to the patient and measures such as talc pleurodesis or indwelling drains can be respectively performed or inserted for fluid management [4].

In recent years, local anaesthetic thoracoscopy (LAT), otherwise known as medical thoracoscopy or pleuroscopy, has become widely used by respiratory physicians as a tool in undiagnosed exudative pleural effusions [4,5]. In the United Kingdom (UK), LAT is widely practised. In 2009, thirty-nine centres were offering the service, with a more recent survey in 2018 by De Fonseka et al. finding that 49 centres were offering LAT regularly to patients (although only 37 responded) [6]. The survey demonstrated access to both rigid and semi-rigid thoracoscopes and wide variability in antibiotic use pre-procedure, use in point of care ultrasound, proceeding in the presence of minimal fluid, use of midazolam and/or fentanyl for sedation and pain relief and doing combined procedures. Some of those specific points will be discussed more in the later paragraphs. These findings are not UK centric, with an India-wide survey showing the same variability in practice [7]. The majority of contemporary practice is based on the now outdated British Thoracic Society 2010 guidelines [4], and in the absence of newer up-to-date publications, narrative reviews such as this can be an invaluable tool. Alternatives to LAT are image-guided biopsies (ultrasound or computed tomogram (CT) directed) with reported sensitivities between 70% and 94%, but there is lack of real time visualisation of tumour, and they do not relieve the patient of breathlessness if the offending effusion is not drained. There is only one direct comparison trial between CT guided biopsy and LAT, and there was a non-statistically significant difference between the two groups for diagnostic rates (CT-guided biopsy 87.5% vs. 94.1% for thoracoscopy) [8].

LAT can thus be performed in the treatment of malignant pleural effusion, pneumothorax and pleural infection. An in-depth discussion of the latter two conditions is beyond the scope of this article, and the article will concentrate on LAT in malignant pleural disease, but we will discuss the other conditions briefly where appropriate. There have been some excellent articles detailing the increasing epidemiology of malignant pleural disease [9,10], and LAT is a crucial part of the pathway [4].

## 2. Indications for Thoracoscopy

### 2.1. Malignant Pleural Disease

LAT is performed predominantly to determine if a malignant process is present in the pleural space. Various case series would suggest that it has a 92.6% diagnostic sensitivity in malignant pleural disease [4,5]. Malignancy in the pleural space often presents with a pleural effusion, and the first diagnostic step is to obtain pleural fluid cytology [10]. However, a positive cytological result is more likely in certain type of cancers (with breast and ovarian cancers being most likely to provide positive pleural fluid cytology) than others such as malignant pleural mesothelioma (MPM) [11,12,13]. In MPM, pleural fluid cytology typically has a low diagnostic yield of 6–32%, and parietal pleura is often required for full characterisation of the tumour [11,12,13]. There is compelling evidence that if the probability of MPM is high, according to clinical and radiological features, a direct to LAT approach should be adopted [14]. One of the unwritten rules of pleural disease is ‘how to obtain a diagnosis and prevent recurrence for my patient in the least possible steps’, and a direct to LAT approach can achieve that. The procedure also provides a simultaneous opportunity for therapeutic interventions including the administration of intrapleural agents such as sterile talc to achieve pleurodesis [4,5,6].

### 2.2. Pleural Infection

LAT has been employed for the treatment of pneumothorax, pleural infection, lung biopsy and sympathectomies [5]. These have been traditionally the domain of cardiothoracic surgery, although it is claimed that the role of formal surgery in pleural disease is reducing [15]. LAT is only feasible in pleural infection at stage 1 and 2, which are the first two stages of pleural infection (simple exudate then fibrinopurulent stage and then an organising stage with pleural peel formation) with division of adhesions, drainage of fluid and placement of a chest tube [15,16,17]. It is not a new concept, but it has not been studied in great detail via large multi-centre randomised trials, nor has there been any recent guidance published on it [18,19]. A recent systematic review of the use of the LAT in pleural infection included eight studies that were all case series or retrospective observational studies, and only two were multi-centre [20]. Whilst the pooled treatment success rate for LAT was 85% (95% CI 80.0–90.0%; I2: 61.8%) when used as first-line intervention or after failure of regular tube drainage or intrapleural therapy, the study designs were poor, and there was a high risk of bias: these factors make advocating LAT for pleural infection difficult. A small randomised clinical trial of 32 patients showed a potential signal towards reduction in length of stay for LAT when compared to chest tube drainage and intrapleural therapy, but these findings are not currently generalisable [17,21,22]. In addition, patients who underwent LAT had a 12.5% higher diagnostic yield from microbiology culture from pleural biopsies taken during the procedure [21]. The recent ‘Studying Pleuroscopy in Routine Pleural Infection Treatment’ (SPIRIT) trial is a multi-centre UK study, which will hopefully provide further results regarding the role of LAT in pleural infection [18]. As an aside, in tuberculous pleural disease, LAT has up to 100% diagnostic sensitivity, but LAT is very resource intensive, and that precludes its use in many countries [23]. A combination of pleural fluid adenosine deaminase, differential cell count and closed pleural biopsy is just as effective in areas of high prevalence of tuberculosis [23].

### 2.3. Pneumothorax

The indication for surgery in those with pneumothorax is predominantly to prevent recurrence, and the whole array of procedures that can be performed are beyond the scope of this article. These are usually completed via video-assisted thoracoscopy or an open thoracotomy approach [24]. However, the use of LAT for pneumothorax is also not a new concept. The largest case series of 124 patients with pneumothorax underwent electrocoagulation of blebs/bullae and talc poudrage pleurodesis under LAT with an average operative time of 15 min [25]. Four (3%) patients required further surgery. However, this is not widely performed. Experienced thoracoscopists can also perform lung biopsy (although this has been almost totally superseded by video-assisted thoracoscopy) [26] and sympathectomy [5]: these are worth a mention but no further discussion, as they are not commonly performed. The authors of this narrative review have never performed these procedures.

### 2.4. Patient Selection

Careful patient selection is required prior to LAT: a detailed history regarding the disease process including previous occupational exposure (e.g., asbestos) and previous malignant disease is important. A baseline functional assessment is useful to assess suitability to proceed with the procedure as well as potential treatment, and therefore, a World Health Organisation Performance Status of 2 or above is recommended [27,28]. The patient’s medical comorbidities may provide important information regarding the patient’s risk factors for the procedure, including drug intolerances and allergies. Antiplatelet therapy (clopidogrel and prasugrel should be withheld for 5 days prior to the procedure, and ticagrelor should be withheld for 7 days prior to the procedure. Aspirin does not need to be stopped. Formal anticoagulation should be withheld for 24 h for therapeutic low molecular weight heparin, 5 days for warfarin and normally 48 h for direct oral anticoagulants (although dagibatran might need to be stopped 4 days before a procedure if the patient’s creatinine clearance is less than 50 millilitres per minute)) and platelet counts should be greater than 50,000 per microlitre of blood and the international normalised ratio should be less than 1.5 [4,27,28,29]. Of note, it is our opinion that whilst platelet count is an established level in the BTS guidance [28,29], platelet activity is also important, and advice from haematology might be obtained prior to a procedure in a thrombocytopenic patient. Prior to LAT, chest radiograph and CT images should be obtained in conjunction with thoracic ultrasonography (TUS) to assess technical suitability and optimal point for thoracoscopy. CT and TUS are essential for providing information on pleural thickening, pleural enhancement as well as adhesions that may impede the procedure, although neither modality can be 100% accurate in excluding adhesions [30,31]. There are few absolute contraindications, including advanced empyema, particularly those with significant adhesions which may make it impossible to insert the thoracoscope safely and fusion of the visceral and parietal pleura in suspected mesothelioma. Relative contraindications include haemodynamic instability, severe hypoxaemia, severe coagulopathies, refractory cough and drug hypersensitivity [4,5].

## 3. Technique, Sedation and Complications

### 3.1. Sedation

The commonest form of sedation is a combination of midazolam and fentanyl, as recommended by the BTS 2010 guidance [4]. Other methods of sedation used include propofol instead of midazolam [32]. Despite the non-inferiority trial of Grendelmeier et al. [32] showing that propofol led to a higher incidence of hypoxaemia and hypotension when compared to midazolam, Tschopp et al. demonstrated that bispectral index guided propofol was a safe method of sedation and facilitated early discharge following the procedure [33]. Other methods of analgesia, including intercostal nerve block, erector spinae plane block or intrapleural lidocaine via semi-rigid thoracoscopes, have been utilised [34,35,36]. There is no standardisation for any sedation method.

### 3.2. Technique

Medical thoracoscopy can either be performed in a surgical theatre or an interventional suite, and there are no specific requirements for the hospital, except that the medical (normally a respiratory physician with an interest in pleural disease or an interventional respiratory physician) and nursing staff are trained at the procedure and can deal with any complications that might arise or refer to a local or regional cardiothoracic unit if urgent surgical intervention is required (in the case, for example, of a significant bleed from intercostal artery rupture. Once sedated and monitored (continuous pulse and oxygen saturations monitoring as well as regular blood pressure monitoring), the patient will lie in the lateral decubitus position, with the side of the pleural effusion to be investigated facing upwards. A point of care USS will be completed pre-procedure to choose a puncture site, and local anaesthetic will be infiltrated at that site. The authors of this article are from two separate centres. In Northumbria Healthcare, between ten and twenty millilitres (depending on patient weight and if an erector spinae block has been performed) of bupivacaine and adrenaline (epinephrine) injection BP 0.5% *w*/*v*, 1 in 200,000 is used. In Leicester NHS Trust, xylocaine (lidocaine 1% + adrenaline 1 in 200,000) is used as standard. A small incision will be then performed with a scalpel, and a Boutin needle (with blunt and sharp ends) will be used to pierce the parietal pleura. Approximately 10–20 spontaneous breaths by the patient are then sufficient to induce a pneumothorax so that the lung ‘peels away’ from the parietal pleura. Then, using small forceps, a tract is then created from the skin to the pleura and a 7-millimetre (mm) trocar is passed through the tract. Fluid can be removed through the trocar using standard suction tubing (due to equalisation of pleural and atmospheric pressure during induction of the above pneumothorax, large amounts of pleural fluid can be removed without the risk of re-expansion pulmonary oedema) to allow for inspection of the pleura and lung with either zero- and fifty-degree scopes, and then biopsy of any lesions deemed appropriate. Talc poudrage can then be performed, and a large bore drain or an indwelling pleural catheter inserted.

Annex S1 shows a step by step guide to the above procedure.

The rigid thoracoscope via a single port is most commonly used by respiratory physicians [6]; however, a few perform LAT with a semi-rigid thoracoscope which bears a resemblance to the more commonly used flexible video bronchoscope [37,38,39,40]. The advantages of the rigid thoracoscope include its ability for good sampling and therapeutic interventions such as breakdown of adhesions and talc poudrage. The semi-rigid thoracoscope may sometimes require a second entry port for better visualisation and has a wider range of view due to its flexibility but acquires smaller sample sizes. This was shown by Rozman et al. where larger samples (24.7 mm^2^) were obtained with rigid forceps than semi-rigid ones (11.7 mm^2^) [39]. Both types of thoracoscopes have similar diagnostic accuracy [37,38,39,40]. Refs. [35,36,37,38] Furthermore, the semi-rigid thoracoscope has a similar safety profile to the fully rigid one [37,38,39,40,41]. Table S1 summarises the salient points in some of the above described studies.

## 4. Complications

LAT is considered a very safe procedure and is described as such by the British Thoracic Society guidelines [4]. Potential major complications occur in 1.8% cases, including empyema, bleeding, post-operative pneumothorax, bronchopleural fistula, pneumonia and port site tumour growth [4,23]. The overall risk of death is 0.1% [4,27,28]. However, this mortality was from earlier case series and was associated with talc-induced respiratory failure due to acute respiratory distress syndrome (ARDS)—non-graded talc was in use at the time. With the now widespread use of graded talc, no further cases of ARDS have been reported. To mitigate against pleural space or skin infections, some centres routinely administer antibiotic prophylaxis [41]. However, there has been no demonstrable difference when Dhooria et al. analysed the effect when patients received a single dose of antibiotic on infection rates [42]. Minor complications include subcutaneous emphysema, minor bleeding, peri-procedure hypotension as well as raised temperature and atrial fibrillation. These occur in 7.3% of cases [4,27,28,29,41].

### 4.1. Comparison to Surgical Video-Assisted Thoracoscopy

Of note, biopsy during LAT has a similar efficacy to video-assisted thoracoscopic surgery (VATS) biopsy in the diagnosis of malignancy, with the latter being performed predominantly under general anaesthesia by thoracic surgeons, although local anaesthetic approaches have been described [43,44]. LAT is less invasive and less expensive than VATS, and it offers an alternative for those who have comorbid conditions and/or are unable to tolerate general (and single lung ventilation) anaesthesia [43,44].

### 4.2. Increasing Diagnostic Yield of LAT

As described above, the diagnostic yield of LAT is very high, but for that small percentage of patients in whom a malignant diagnosis is suspected (those with high pre-test probability for example with red flag symptoms and pleural nodules and masses on imaging) and whose biopsy is interpreted as benign (inferring a false negative biopsy), further procedures (surgical video-assisted thoracoscopy or image-guided biopsies) are often required [41,43,44,45]. Anecdotally, this creates significant patient anxiety and adds delays in any potential treatment pathway, which can also be exacerbated by other factors such as delay in pathology processing [45]. High pre-test probabilities for malignancy at LAT are associated with pleural masses, nodules, thickening, and irregularity on contemporaneous radiological imaging with reported sensitivities of 36–51% and specificity between 88 and 100% [46,47].

False negative results are often thought to be due to ‘difficult’ LAT, where the amount of fluid might be minimal, the lung might not ‘go down’ at pneumothorax induction resulting in limited views, or where there might be significant loculations or adhesions again limiting access to the parietal pleura or where ‘deep’ (incorporating adipose and muscle tissue) biopsies of the parietal lining were not performed or feasible [48].

It is important to note the distinction between a true false negative biopsy and pathological findings that would suggest non-specific pleuritis (NSP) which describes chronic pleural inflammation which does not have a specific benign or malignant cause. Up to 30% of cases of exudative pleural effusions can have NSP as a final diagnosis. However, close clinical and radiological follow-up is required for a period of 24 months due to the risk of malignant transformation [48].

There are thus a number of suggested practices to improve diagnostic yield, as the macroscopic appearance of the pleura is neither sensitive nor specific [49].

Firstly, rapid onsite evaluation (ROSE) of the specimens by histopathologists in real time at the time of LAT is an option, notwithstanding the limitations in assessing some cancers such as mesothelioma where differentiation between benign fibrinous material, atypical mesothelial cells and tumour with ROSE might prove impossible. ROSE has not been tested in large multi-centre trials, but monocentric observational data would suggest that combining radiological imaging and ROSE findings would increase diagnostic yield [50]. However, widespread applicability is challenging, as having a histopathologist or a cytotechnologist presents at all LAT would not be possible given that, for example, in the United Kingdom at least, there is a lack of histopathologists or cytotechnologists [51].

A new, exciting development recently presented at the 2020 and 2021 European Respiratory Society Congress is confocal laser endomicroscopy [52]. This is an optical imaging tool used at endoscopy which provides live in vivo images of tissues and detects malignant cells. The largest case series of 62 patients accurately described normal (100% positive predictive value (ppv) for the ‘full chia seed sign’ suggesting benign pathology) and malignant pathology (75% ppv for abnormal tissue architecture and 68% for dysplastic vessels). Again, larger, multi-centre studies are required for the external validation of those findings [52]. In a similar vein, narrow band imaging (NBI) and autofluorescence, techniques which light up abnormal tissues and enable targeted biopsies, have been suggested, but widespread use is dependent on training and resources [53,54,55].

Supplementary Table S1 provides a summary of the some of the relevant papers so far. 

### 4.3. Combined Procedures at the Time of LAT

Pleurodesis is commonly performed if malignancy is strongly suspected [4,5,6]. This is an important aspect of pleural fluid management, but if pleurodesis is performed, further interventional procedures might prove very hard, and talc might show up as abnormal hypermetabolic areas on CT or positron emission tomogram (PET) scans [56]. This comes back to the above point where some centres advocate ROSE preparation at the time of biopsy [50]. Pleurodesis at thoracoscopy should also be informed by patient-centred management, as Bhatnagar et al. showed that in the absence of non-expandable lung, talc poudrage at thoracoscopy had no difference in the rate of pleurodesis at 90 days when compared with talc slurry through a chest tube [57]. One could argue that if one is performing a LAT for diagnosis as well as fluid control, then pleurodesis at the same time prevents a further procedure, but pleurodesis via LAT should not be offered as a superior choice.

There has been a palpable shift, evidenced by case series [58], towards day case LAT with indwelling pleural catheter (IPC) insertion in the last few months, which has been bolstered by the pleural guidance written by the British Thoracic Society at the start of the COVID-19 pandemic [59]. A trial is underway in the United Kingdom looking at the merits of day case LAT with IPC insertion (and aggressive subsequent drainage via the IPC) versus standard care (large bore drain, pleurodesis and admission) [60].

There is also the possibility of conducting another procedure concurrently (such as endobronchial ultrasound) if the ROSE preparation suggests a non-malignant process in a patient with high index of suspicion for malignancy [61]. This is not the norm in the vast majority of centres, and it would again have significant resource implications.

## 5. Conclusions

For those presenting with an unexplained pleural effusion, with a high pre-test probability of malignancy, LAT is a safe procedure with high diagnostic sensitivity. Guidance governing its practice is outdated, but updated documents from the British Thoracic Society and European Thoracic Society are expected in late 2022. We hope that the above narrative review informs the general physician about a cornerstone procedure in pleural disease and that for established respiratory physicians, it serves as an expert opinion-based summary of the literature.

### 5.1. Future Directions

We highlight areas for future research, which are:

- The feasibility and safety of day case LAT with IPC insertion;
- The widespread application of new techniques such as ROSE preparation and confocal laser endomicroscopy;
- The feasibility of LAT in early stage pleural infection (with or without intra-pleural fibrinolytics) compared with surgical techniques.

### 5.2. Main Messages

- Medical thoracoscopy is a diagnostic and therapeutic procedure for unexplained pleural effusion with a very high diagnostic yield.
- In those patients with a high pre-test probability for cancers such as mesothelioma, a direct to biopsy approach via medical thoracoscopy should be the preferred approach.
- Medical thoracoscopy has an acceptable safety profile, the main concerns being pleural infection (empyema) and prolonged air leaks.

## Data Availability

Not applicable.

## Supplementary Materials

The following supporting information can be downloaded at: https://www.mdpi.com/article/10.3390/healthcare10101978/s1, Annex S1 is a pictorial representation of the procedure. Table S1 is a summary of the relevant papers discussed in the paper.
