# Supplementary material for: Local Anaesthetic Thoracoscopy for Pleural Effusion—A Narrative Review
Source: Healthcare (Basel). 2022 Oct 9;10(10):1978. doi: 10.3390/healthcare10101978 (PMC9601808; doi:10.3390/healthcare10101978)
Supplement: Supplementary file 1 [file healthcare-10-01978-s001.zip › Annex S1.pdf]

Thoracoscopy

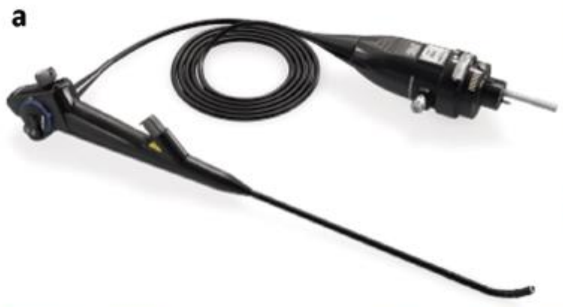

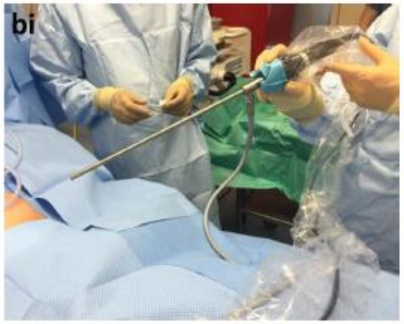

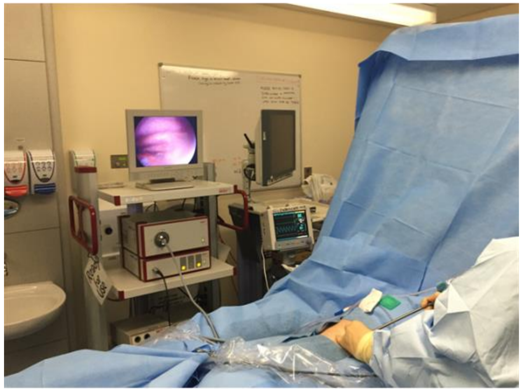

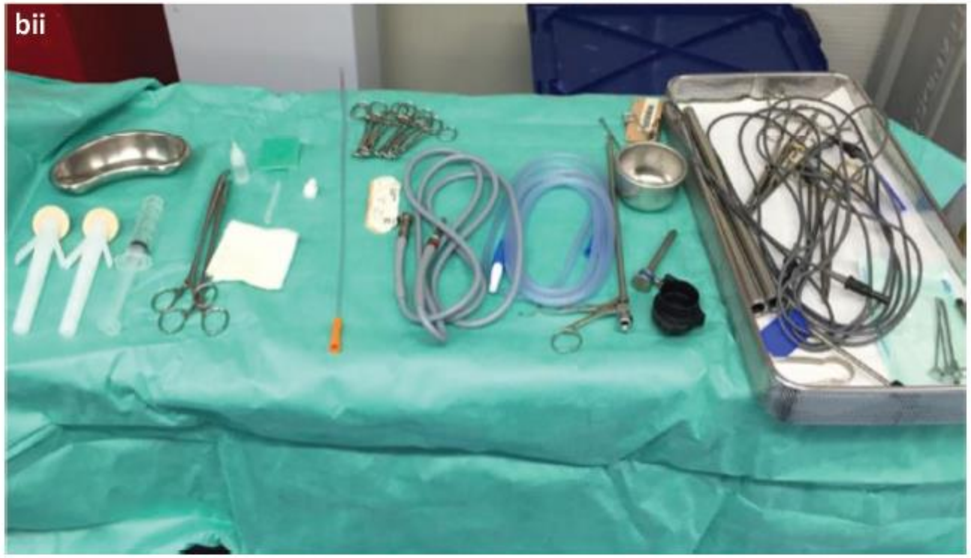

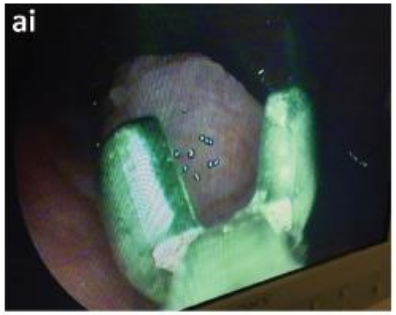

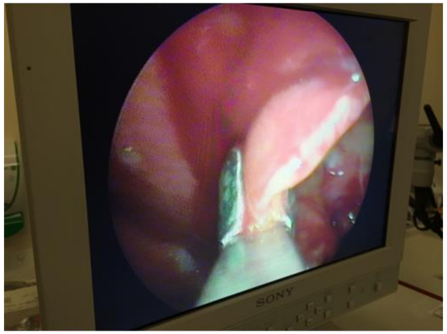

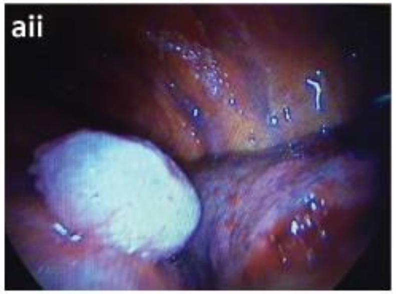

Talc poudrage

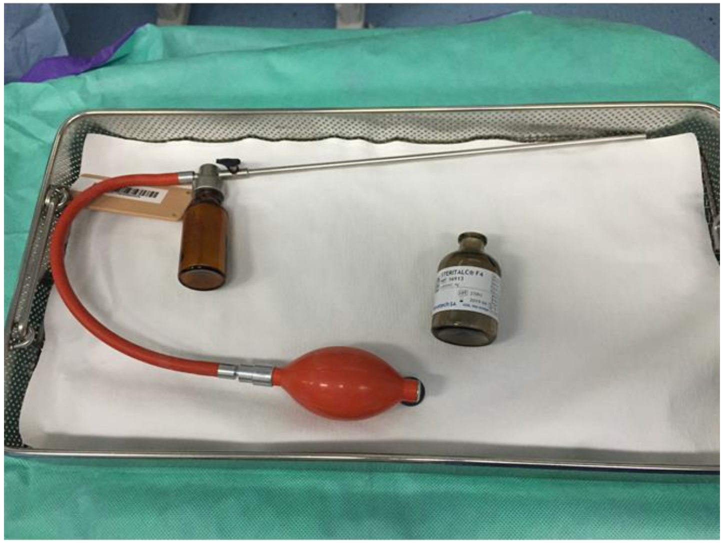

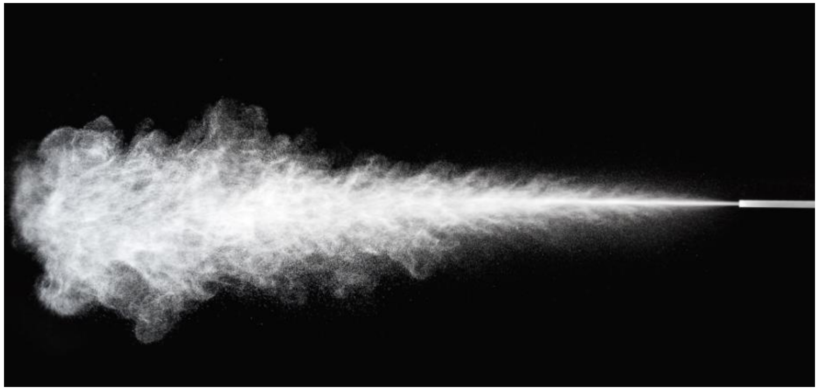

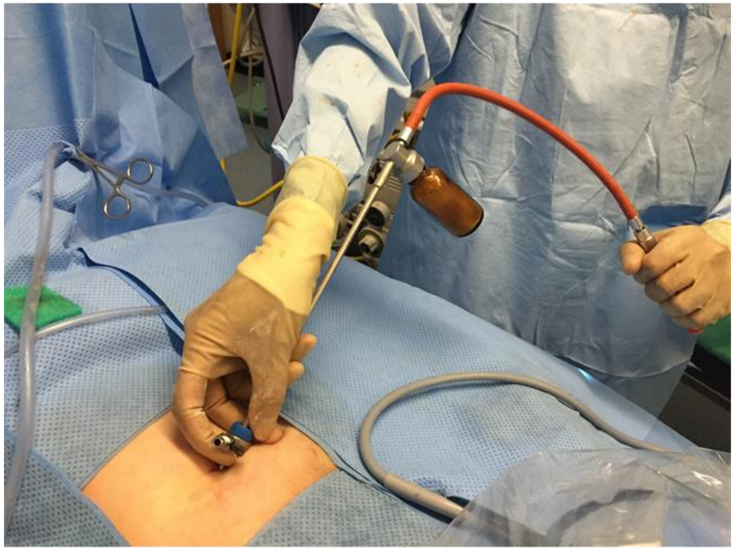

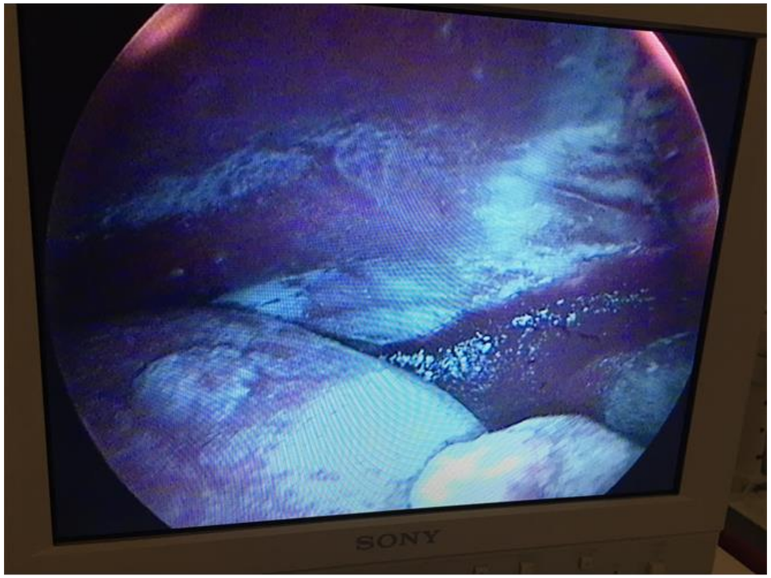

Pre-procedure Imaging

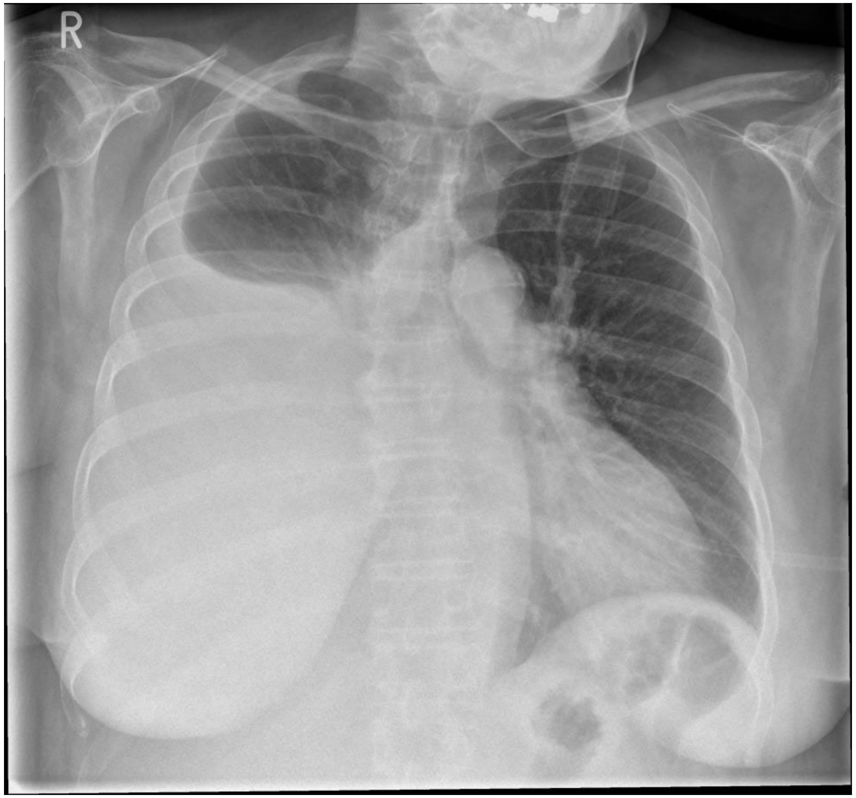

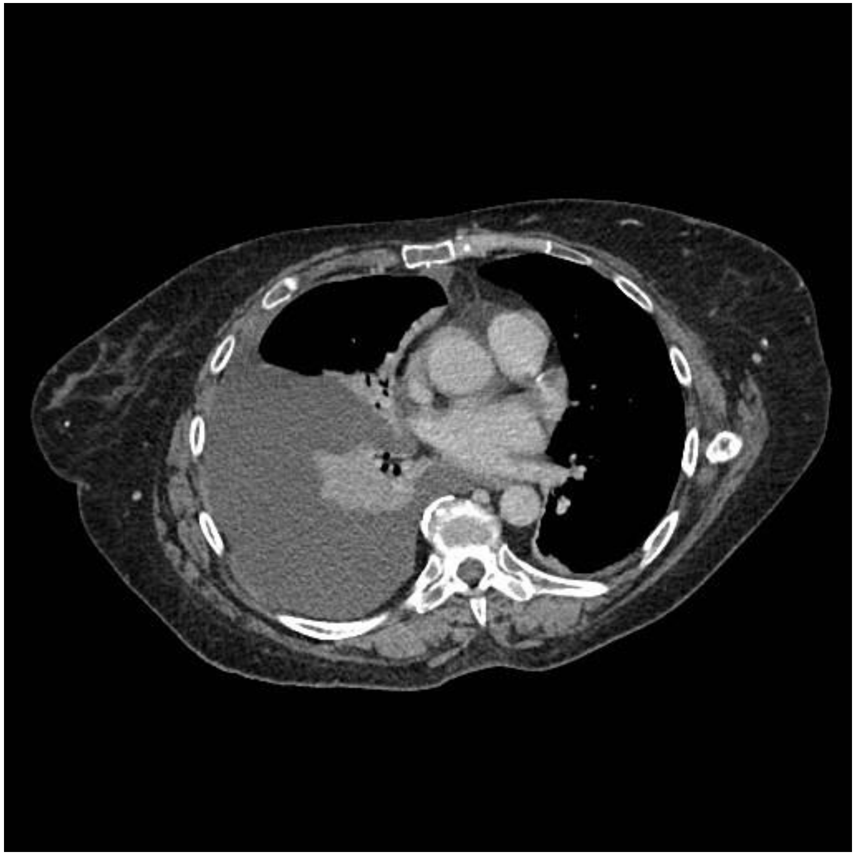

Step by Step Thoracoscopy

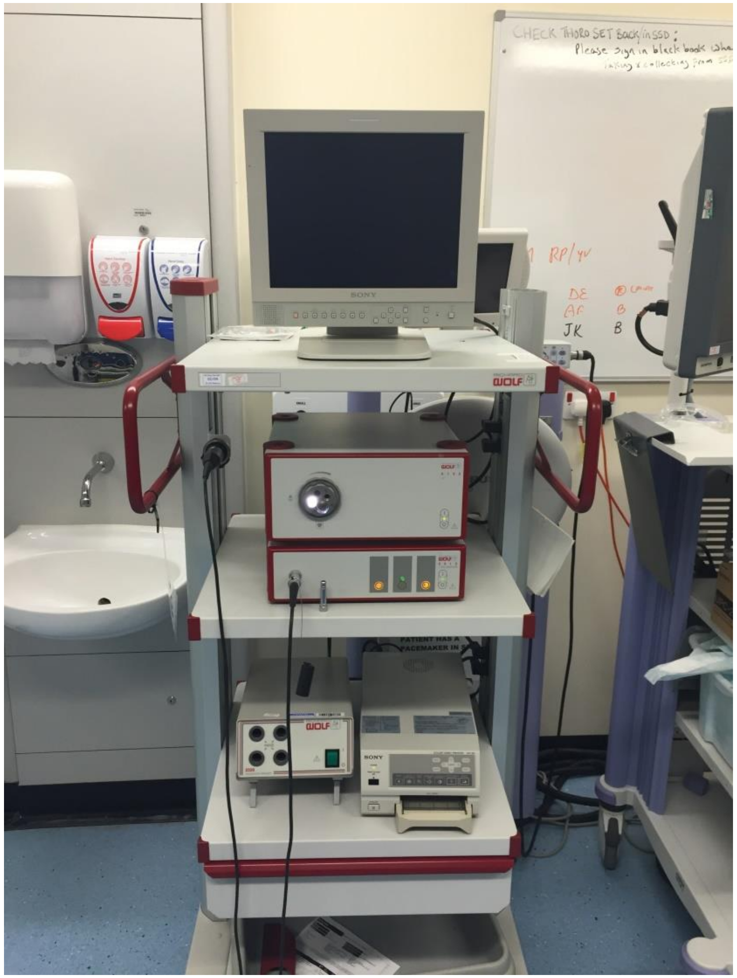

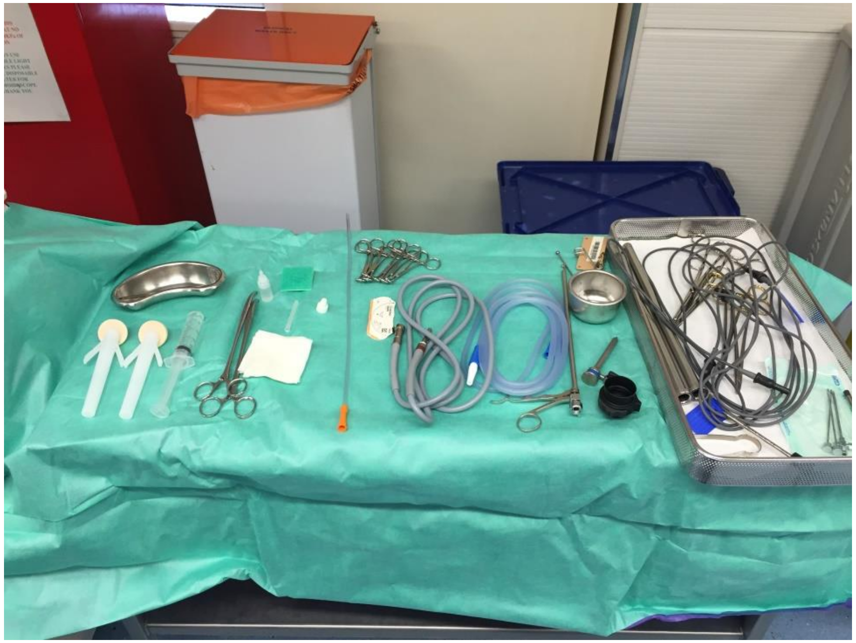

Monitoring & USS

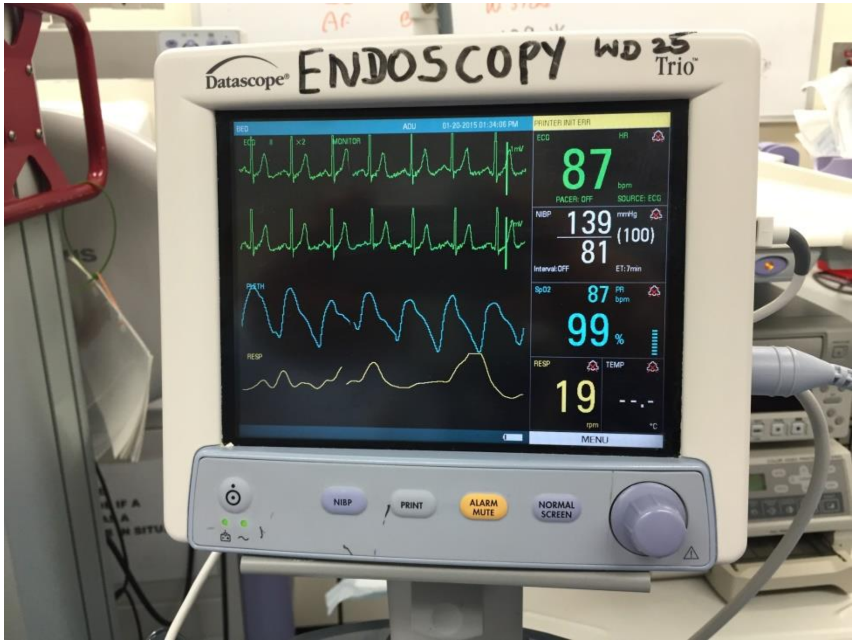

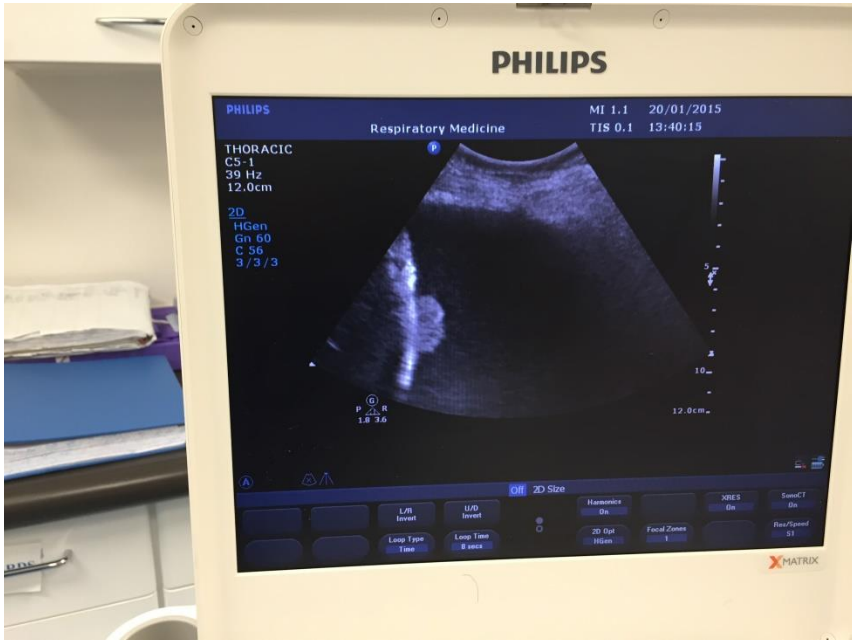

Sedation & Local anaesthesia

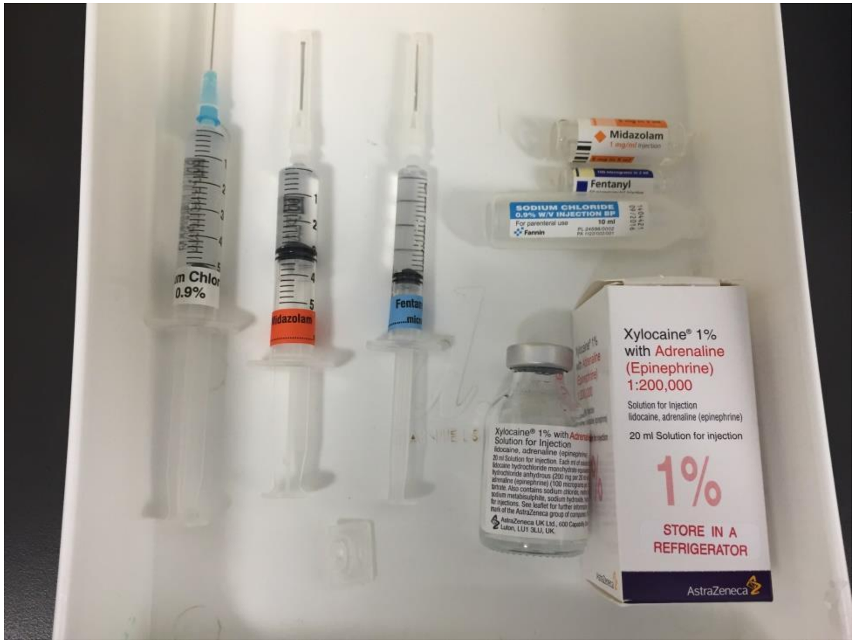

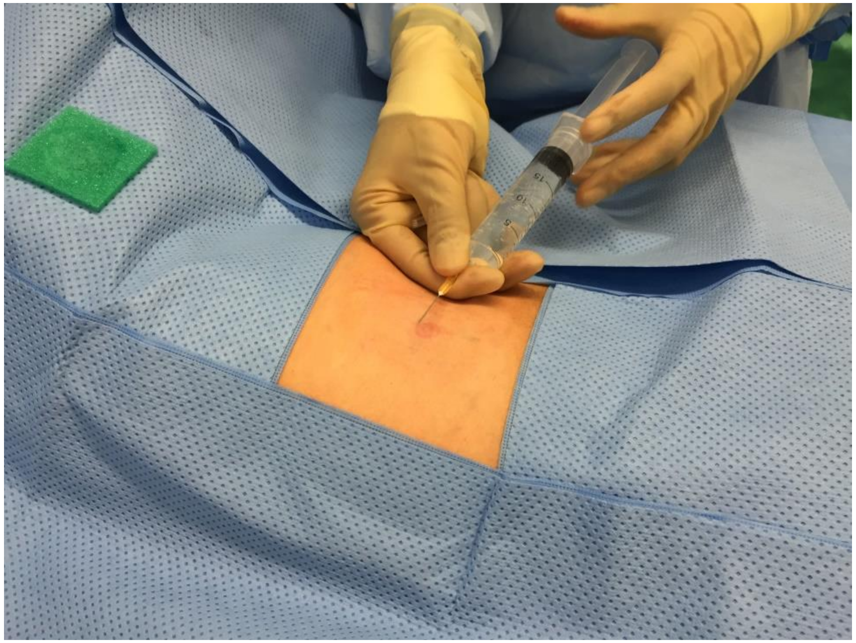

Aspiration & equipment prep

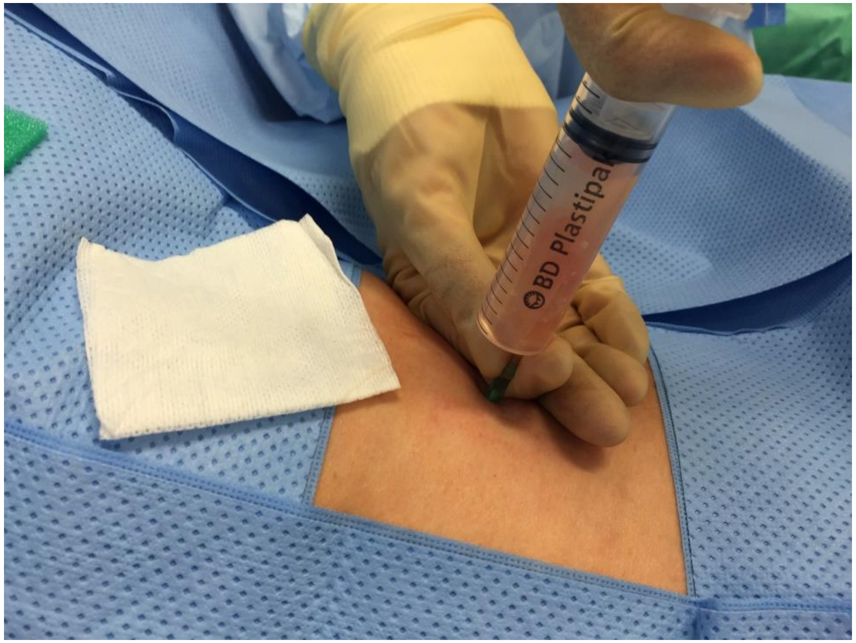

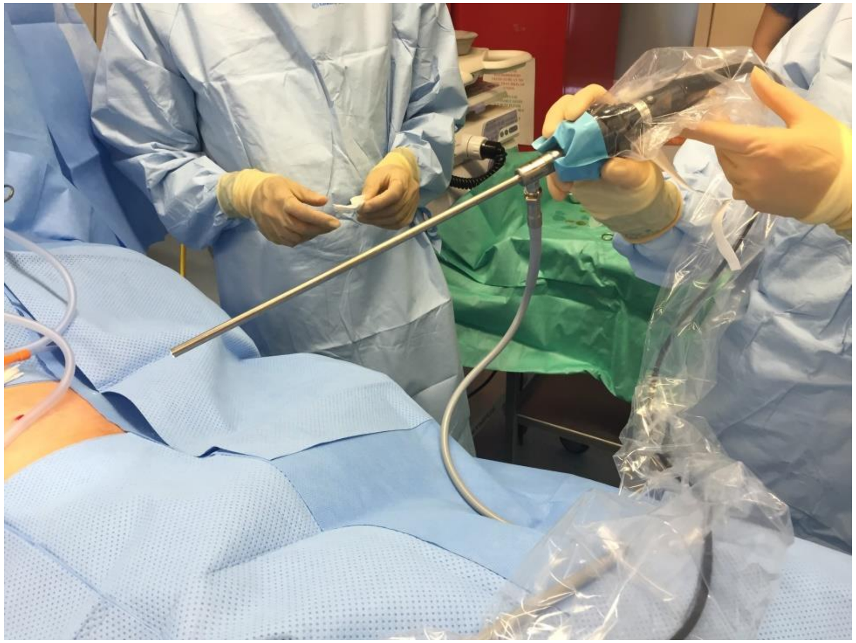

Incision & blunt dissection

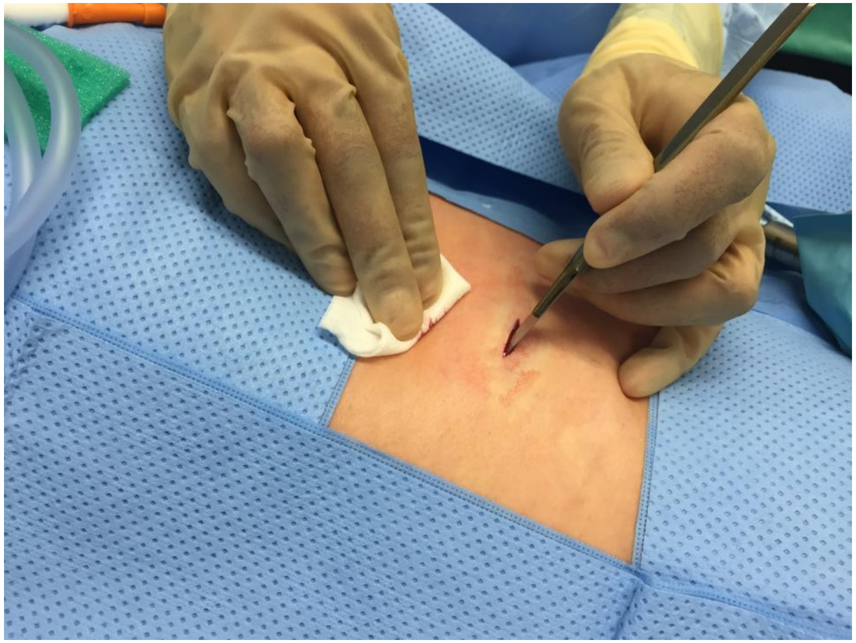

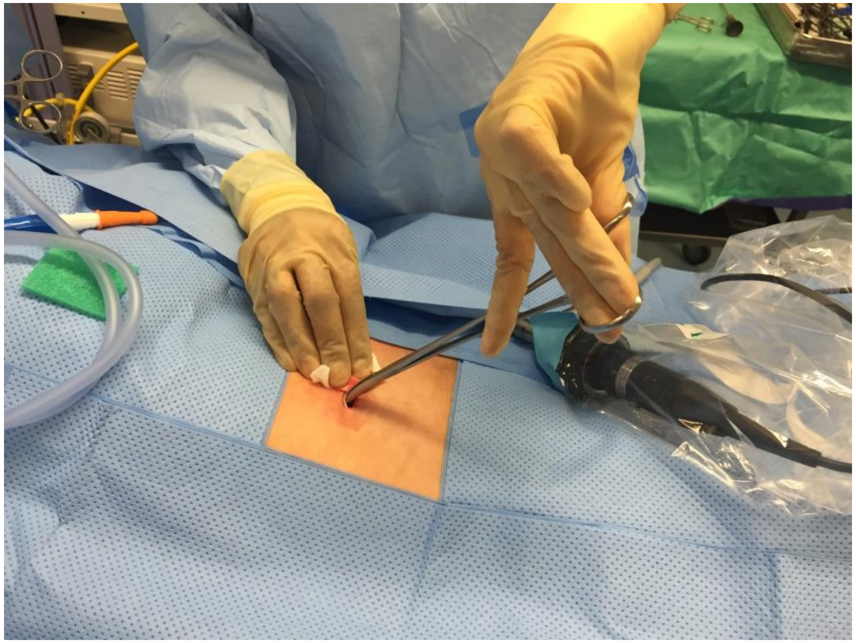

Trocar & cannuale with valve

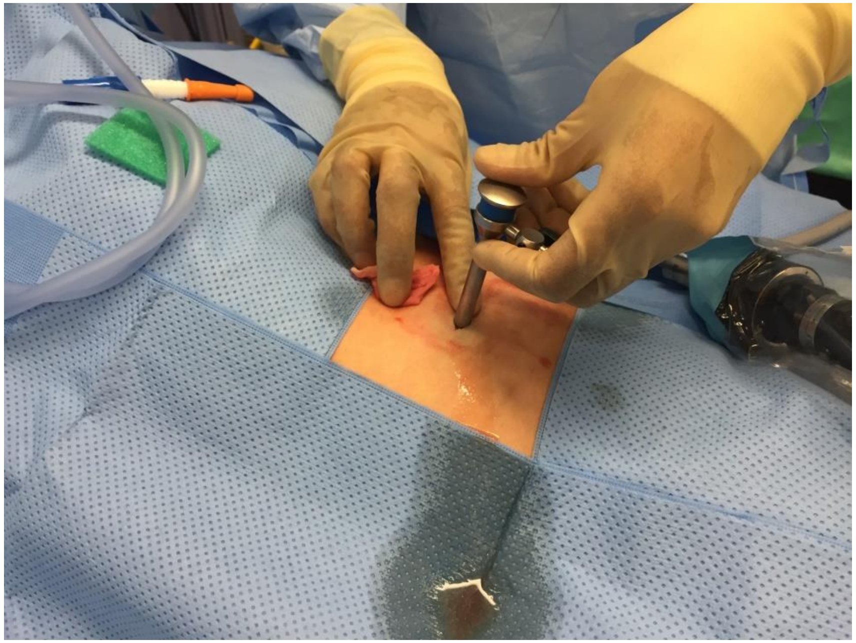

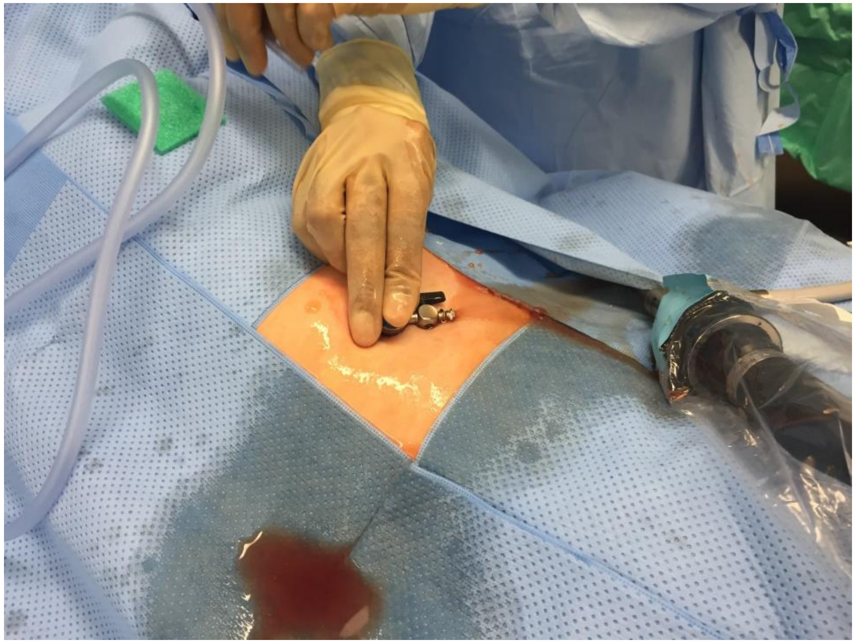

Suction

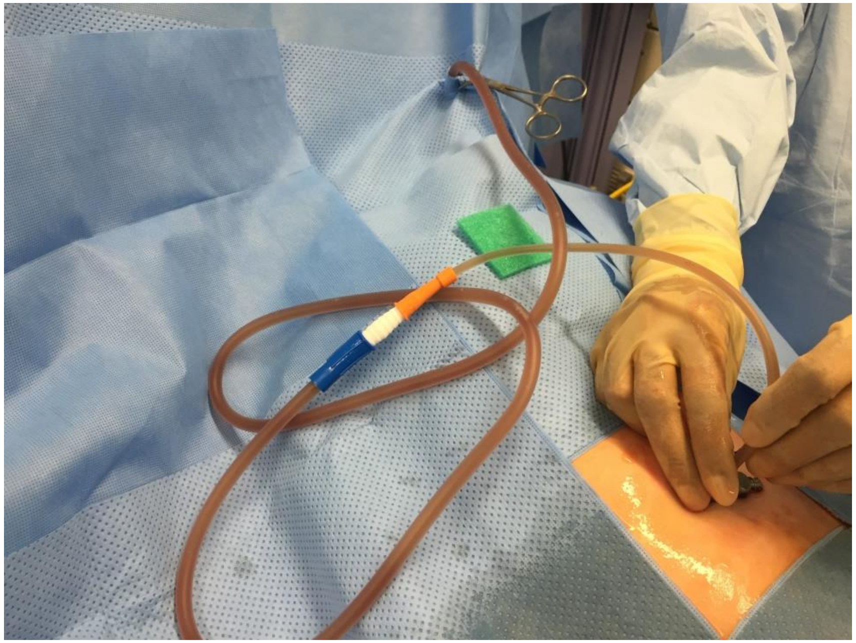

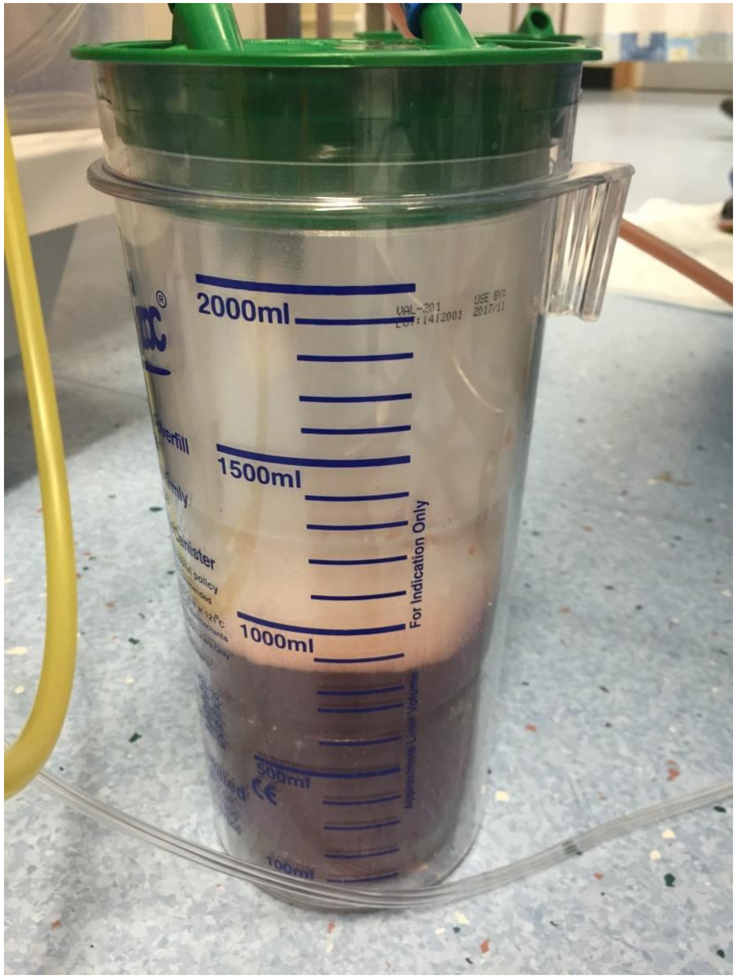

Camera

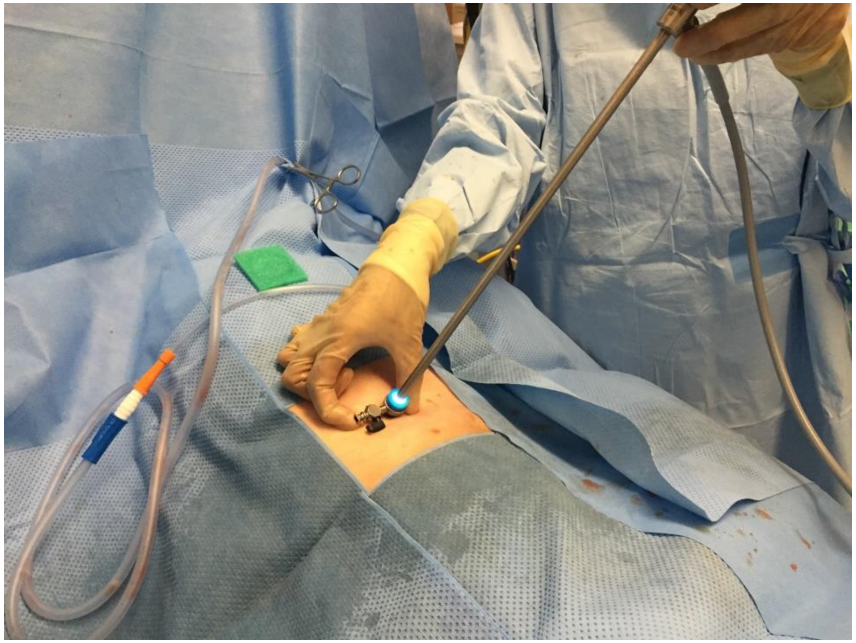

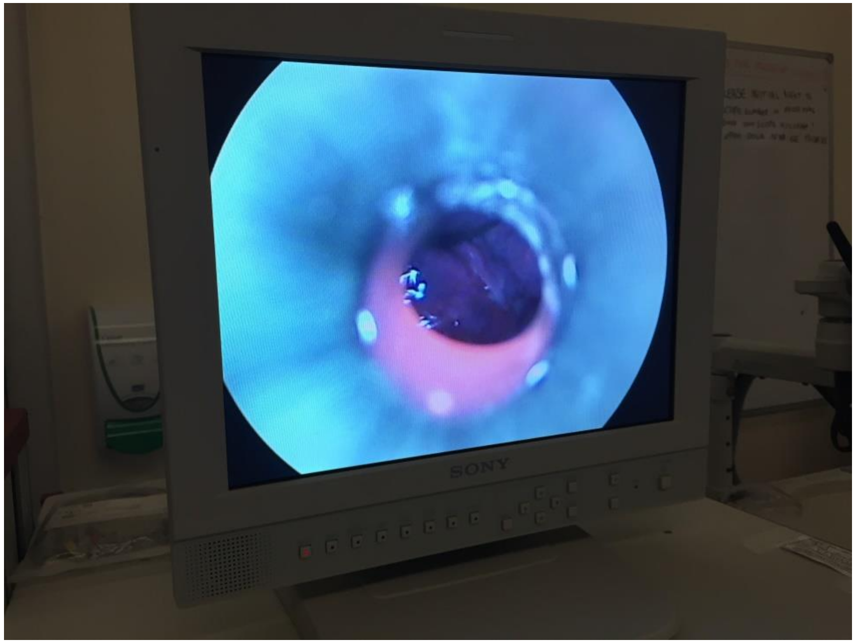

Macroscopic pathology

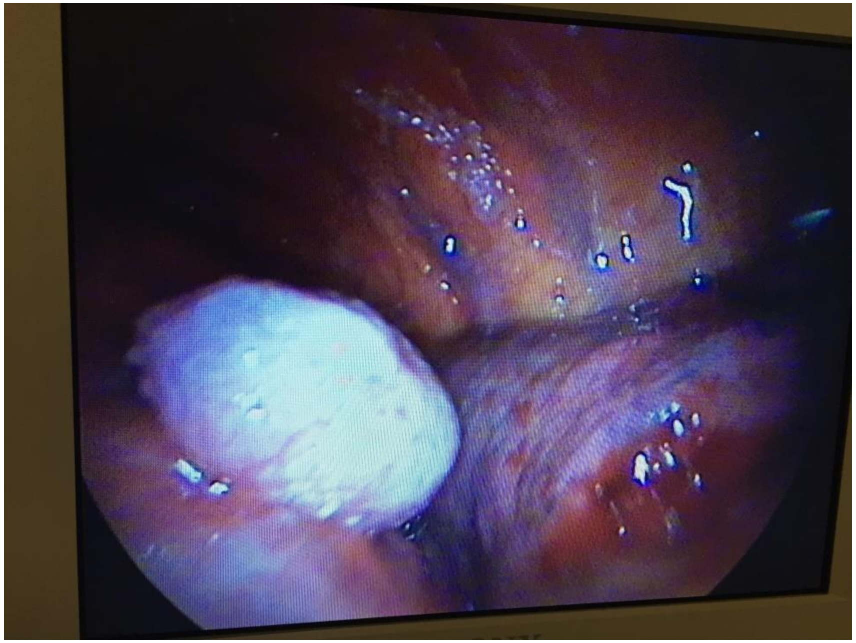

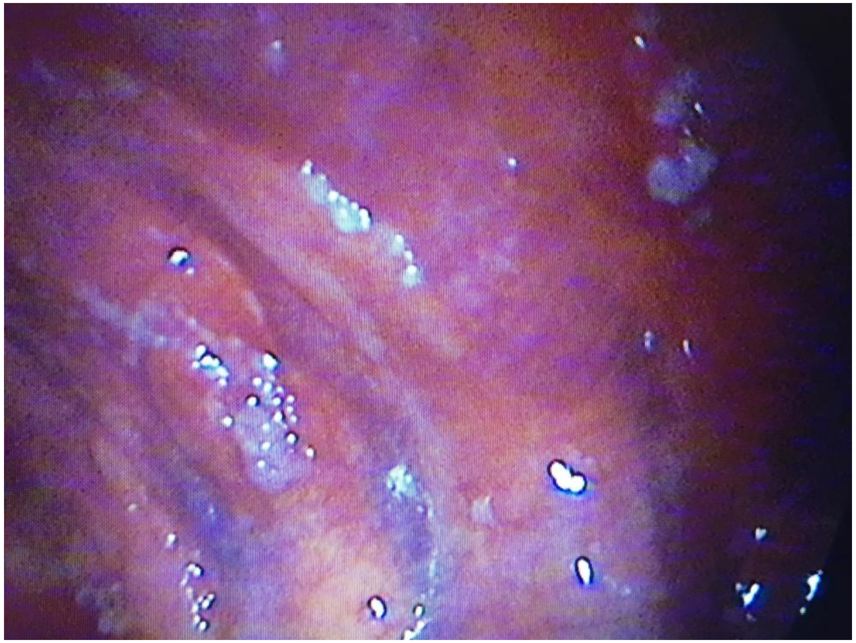

Room set-up & biopsy forceps

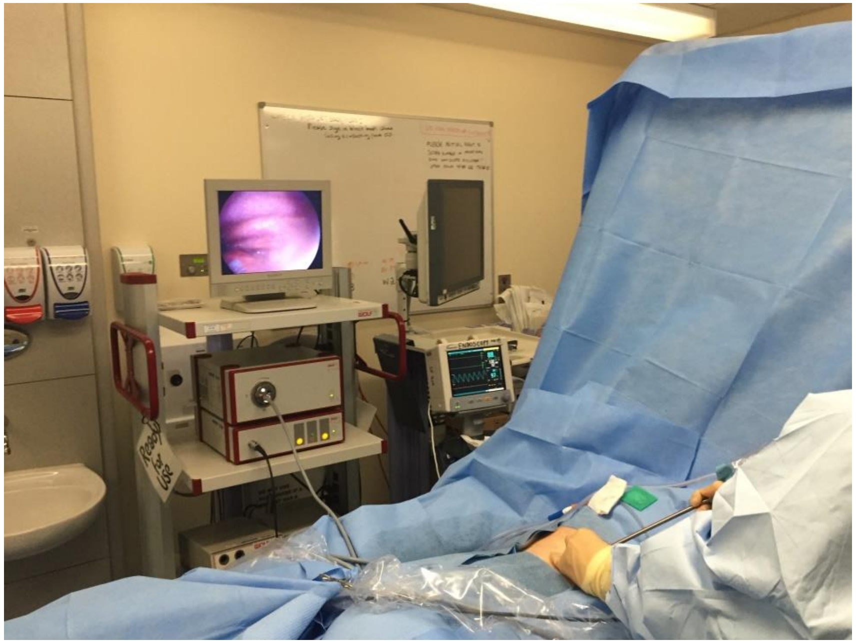

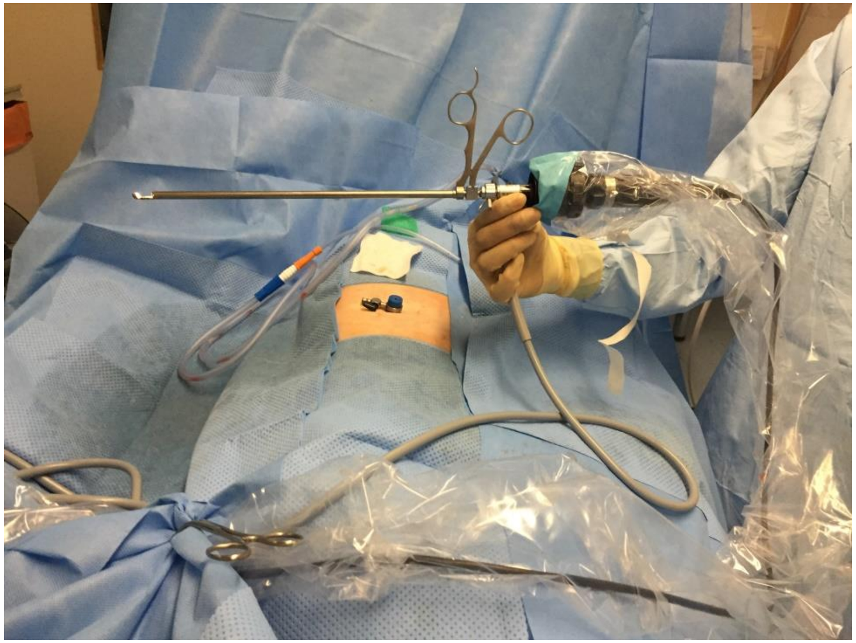

Biopsies

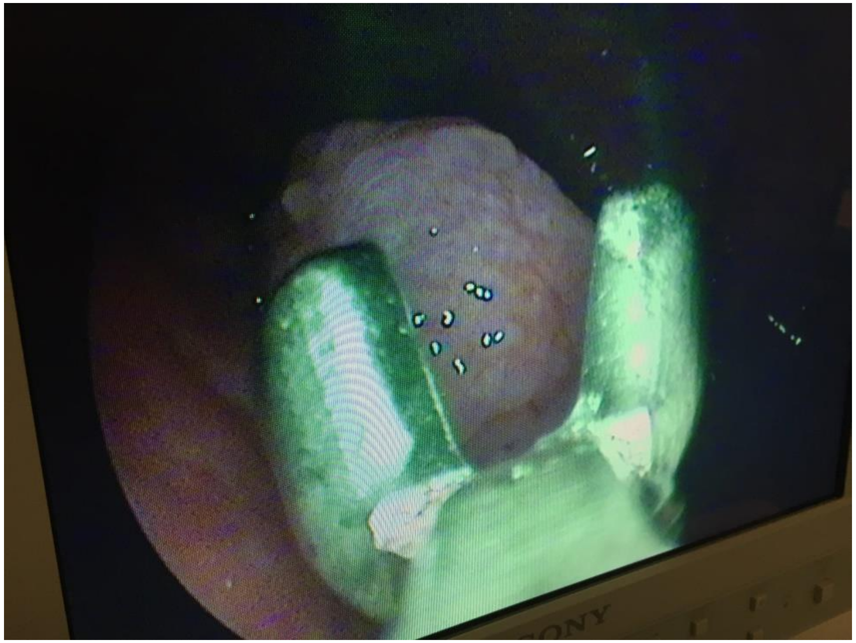

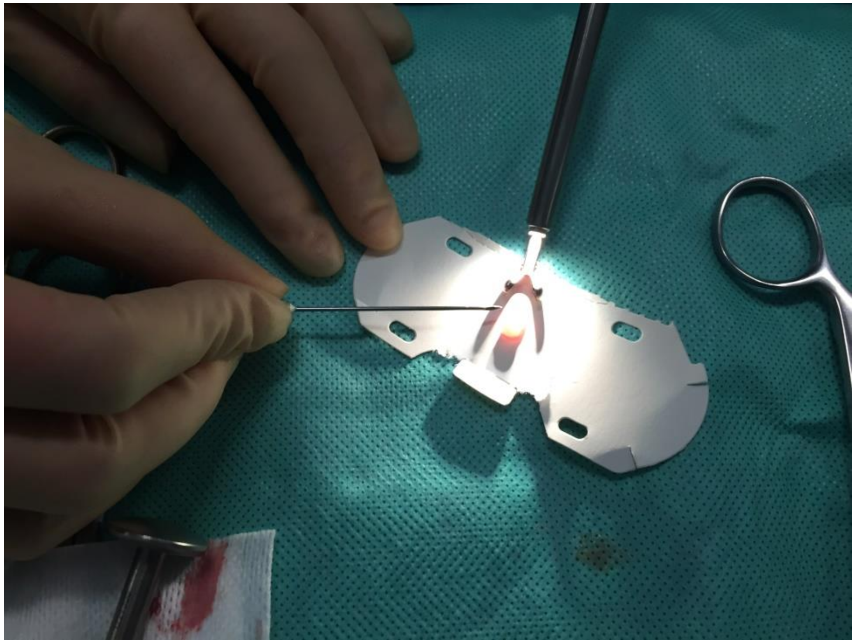

Biopsies + talc poudrage kit

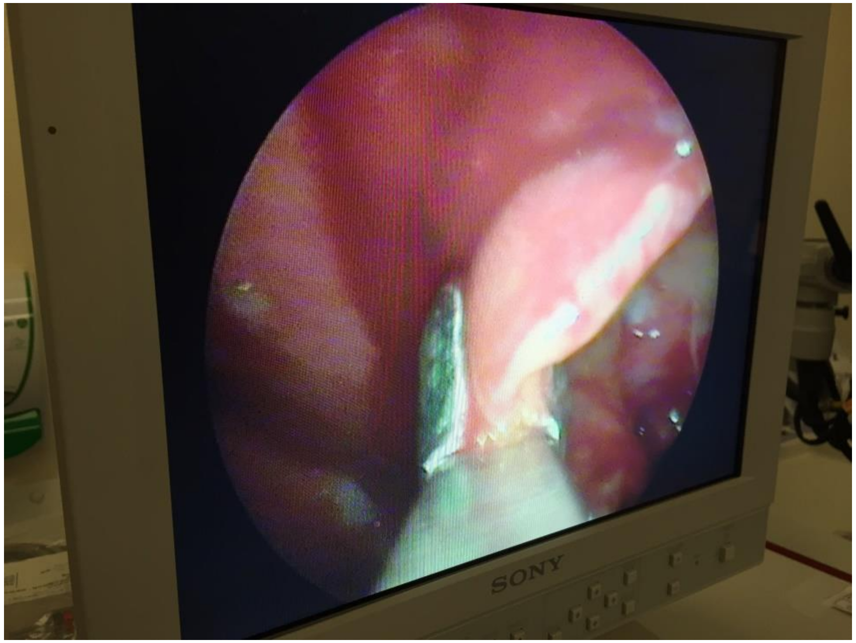

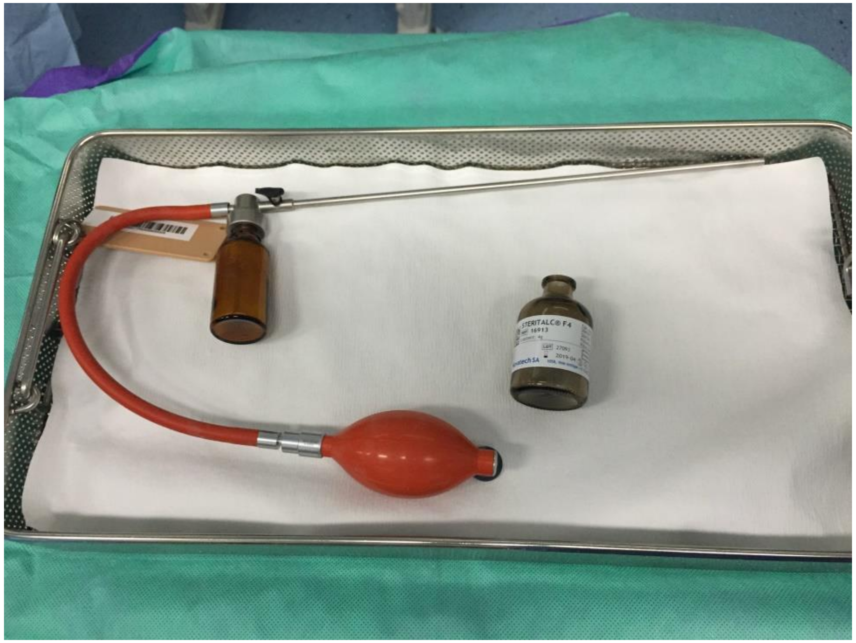

Talc poudrage

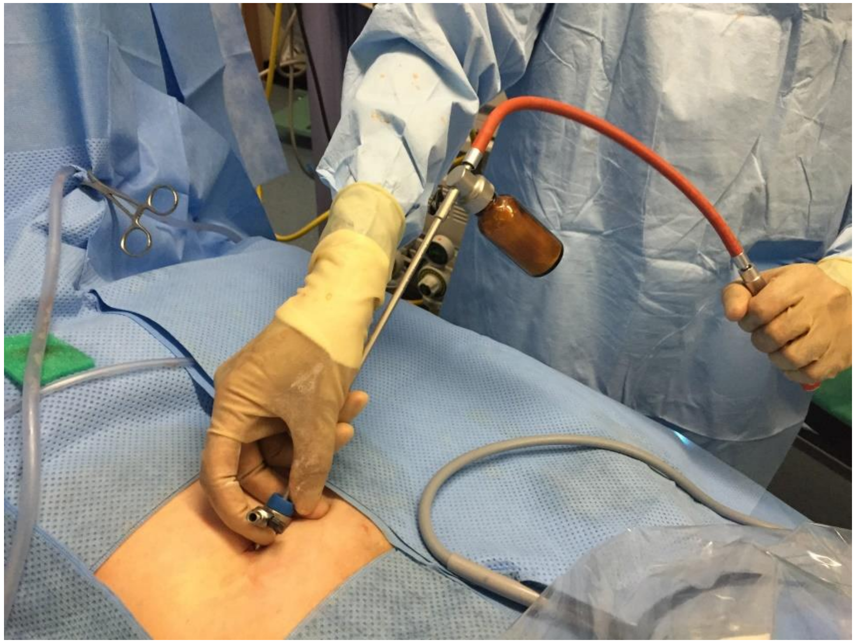

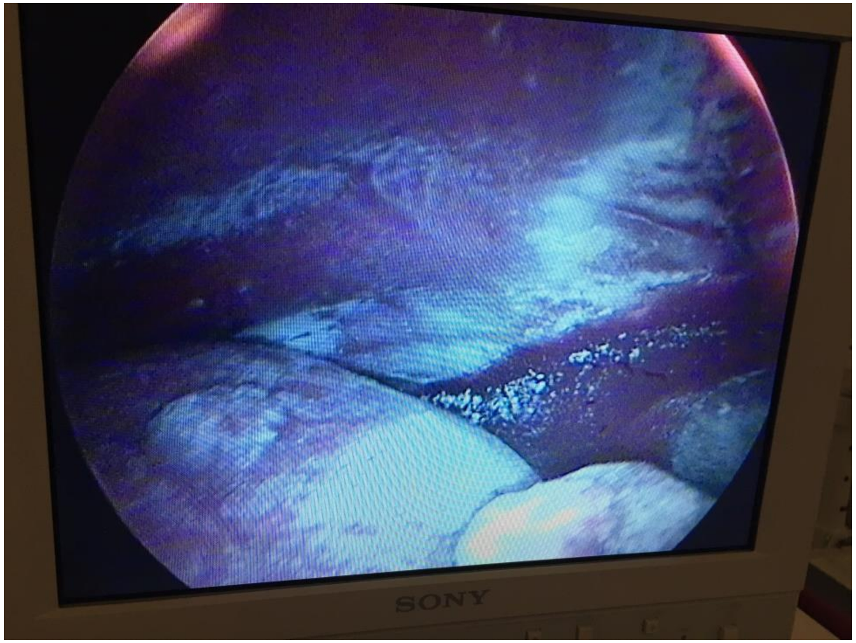

Talc poudrage + wide bore drain

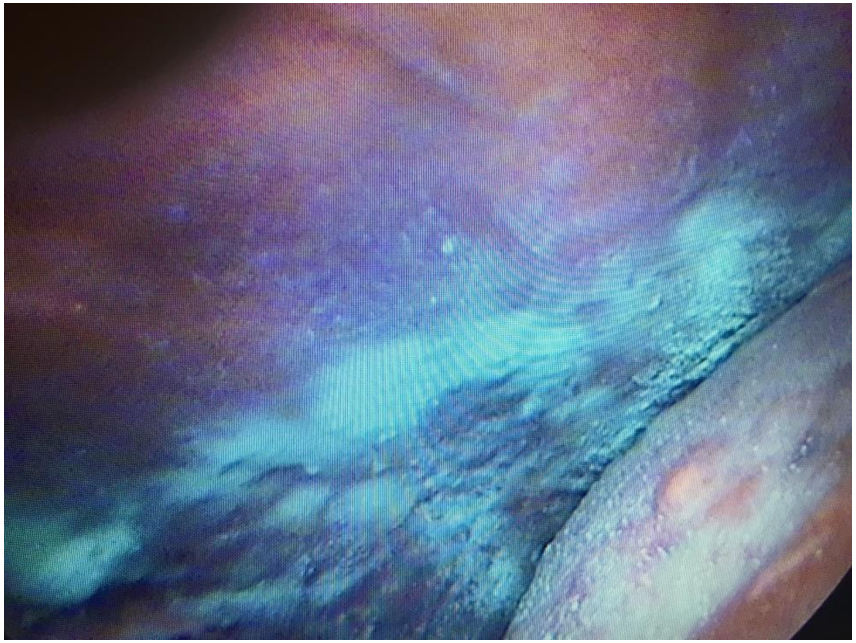

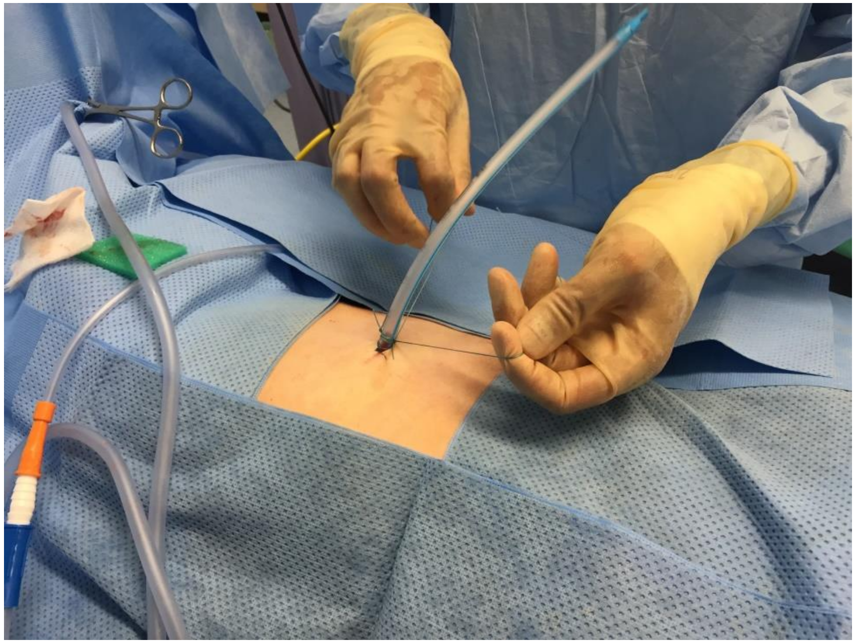

Dressing & drain

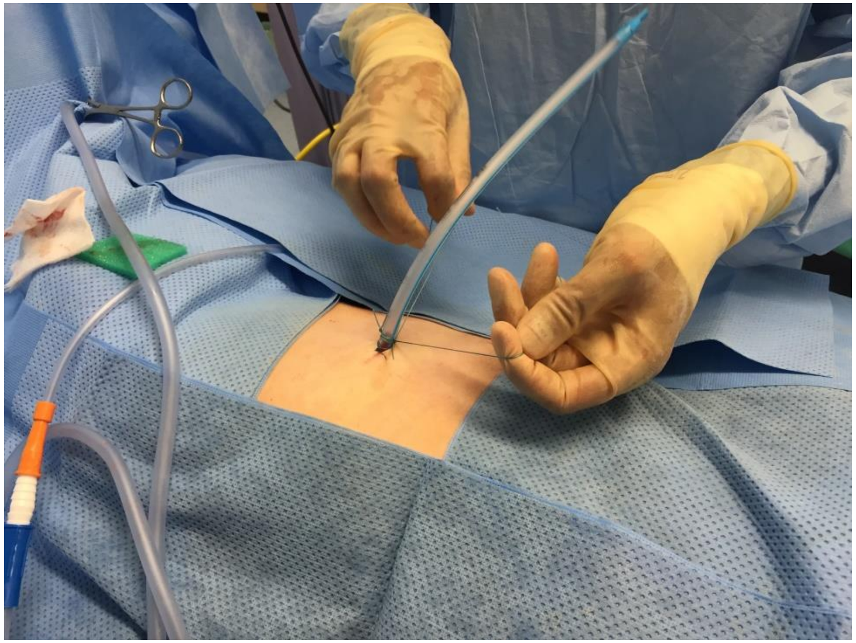

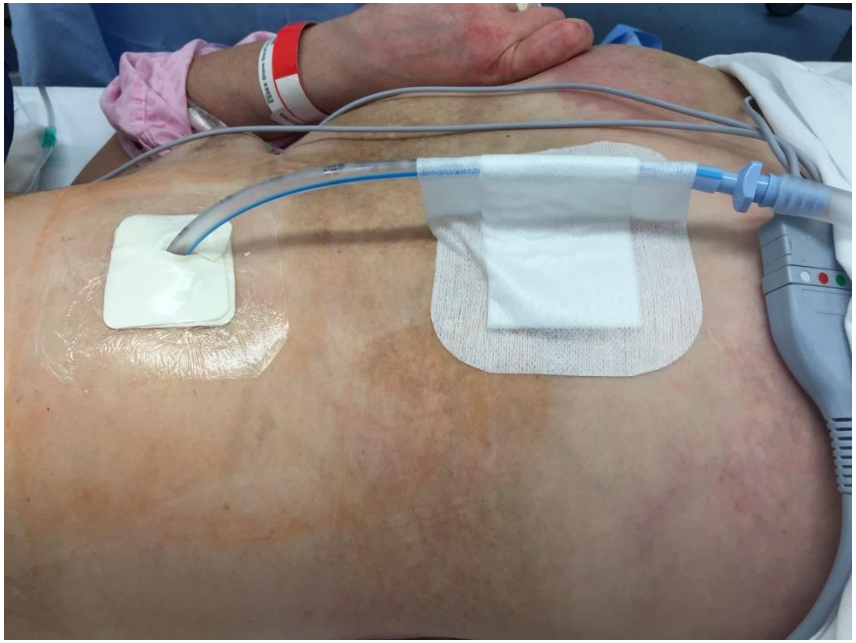

Post-procedure imaging

1 hour post LAVAT

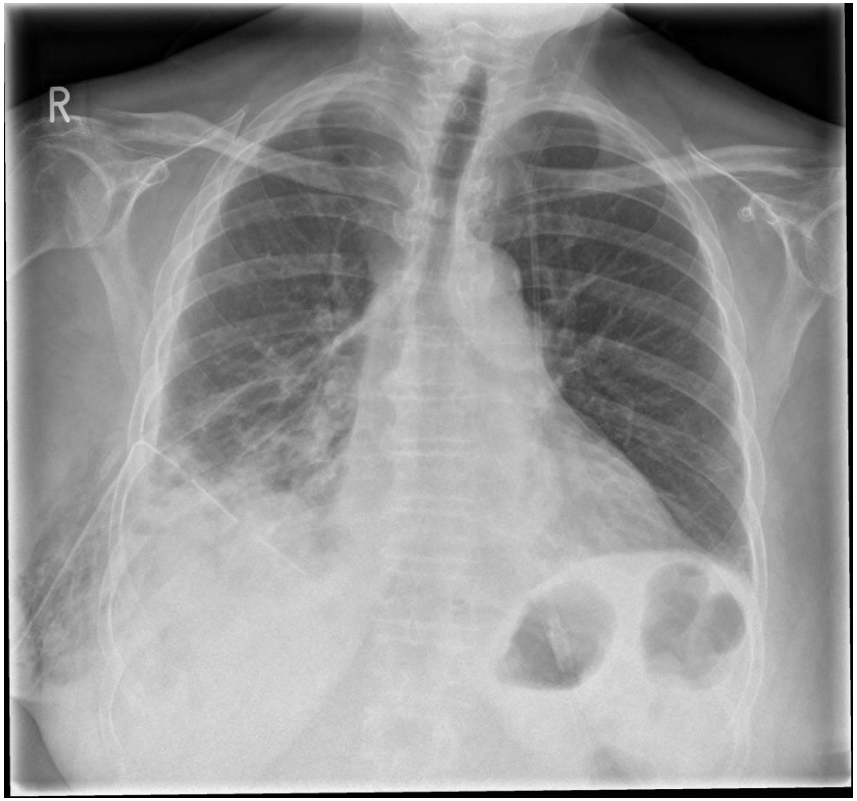

1 week follow-up

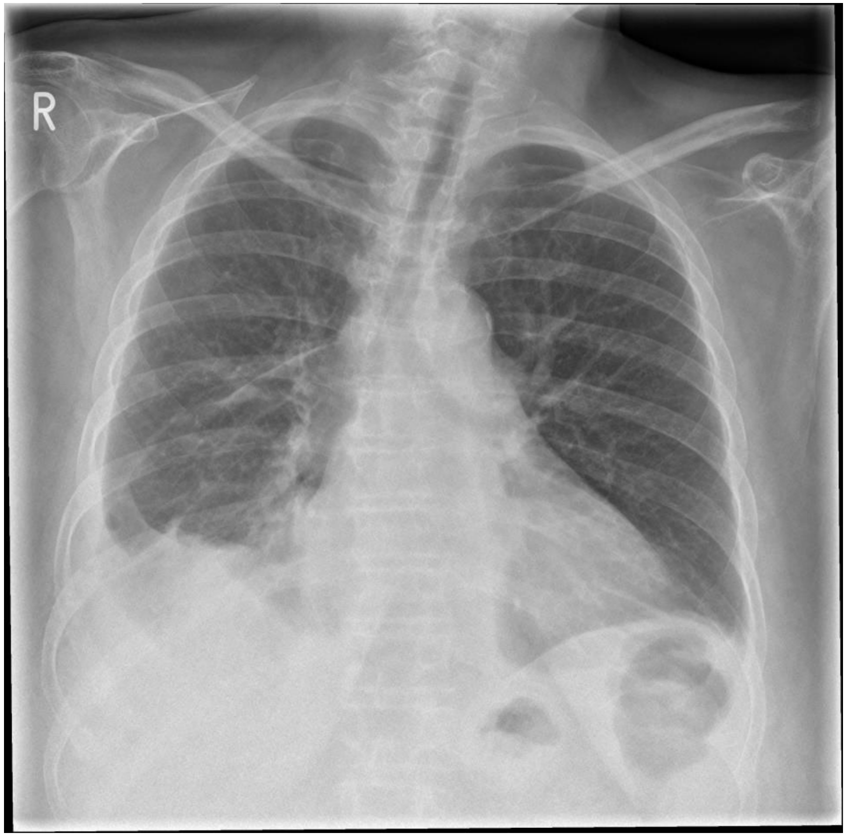

Diagnosis: High Grade Serous Adenocarcinoma - Ovarian
